# The activity of a PI3K δ-sparing inhibitor, MEN1611, in non-small cell lung cancer cells with constitutive activation of the PI3K/AKT/mTOR pathway

**DOI:** 10.3389/fonc.2023.1283951

**Published:** 2023-11-14

**Authors:** Giuliana Papoff, Dario Presutti, Valentina Fustaino, Andrea Parente, Clelia Calandriello, Stefano Alemà, Ferdinando Scavizzi, Marcello Raspa, Giuseppe Merlino, Massimiliano Salerno, Mario Bigioni, Monica Binaschi, Giovina Ruberti

**Affiliations:** ^1^ Institute of Biochemistry and Cell Biology, National Research Council (IBBC-CNR), Monterotondo, Rome, Italy; ^2^ European Mouse Mutant Archive (EMMA), INFRAFRONTIER, Monterotondo Mouse Clinic, IBBC-CNR, Monterotondo, Rome, Italy; ^3^ Menarini Ricerche S.p.A., Rome, Italy

**Keywords:** NSCLC, PI3K inhibitor, EGFR-TKIs, drug-resistance, combination therapy

## Abstract

**Background:**

Lung cancer remains the leading cause of cancer-related death worldwide. Targeted therapies with tyrosine kinase inhibitors (TKIs) result in improvement in survival for non-small cell lung cancer (NSCLC) with activating mutations of the epidermal growth factor receptor (EGFR). Unfortunately, most patients who initially respond to EGFR-TKI ultimately develop resistance to therapy, resulting in cancer progression and relapse. Combination therapy is today a common strategy for the treatment of tumors to increase the success rate, improve the outcome and survival of patients, and avoid the selection of resistant cancer cells through the activation of compensatory pathways. In NSCLC, the phosphoinositide-3-kinase/protein kinase B (AKT)/mammalian target of rapamycin (mTOR) pathway has been heavily implicated in both tumorigenesis and the progression of disease.

**Objectives:**

In this study, we investigated the efficacy of a PI3K δ-sparing inhibitor, MEN1611, in models of NSCLC sensitive and resistant to EGFR inhibitors (erlotinib and gefitinib) with a wild-type *PIK3CA* gene.

**Methods:**

We performed functional, biochemical, and immunohistochemistry studies.

**Results:**

We demonstrated good efficacy of MEN1611 in NSCLC devoid of *PIK3CA* gene mutations but with constitutive activation of the PI3K/AKT pathway and its synergistic effect with gefitinib both *in vitro* and *in vivo*.

**Conclusions:**

Overall, this preclinical study indicates that the inhibitor could be a candidate for the treatment of NSCLC with an erlotinib/gefitinib-resistant phenotype and constitutive activation of the PI3K/AKT pathway, a phenotype mimicked by our model system.

## Introduction

Lung cancer remains the most common cause of cancer-related death in the West (1). Non-small cell lung cancer (NSCLC) harboring activating mutations in the epidermal growth factor receptor (EGFR) kinase domain may be treated with EGFR–tyrosine kinase inhibitors (TKIs), including erlotinib and gefitinib. However, most patients who initially respond to this treatment ultimately develop resistance to therapy, resulting in cancer progression and relapse (2, 3).

We have developed a model system of cell lines resistant to inhibitors of EGFR to identify and characterize mechanisms of resistance to target therapy and to investigate preclinical protocols of combination therapy *in vitro* and *in vivo* (4, 5). The PI3K/AKT/mTOR signaling pathway, which controls multiple cellular processes including metabolism, motility, proliferation, growth, and survival, is one of the most frequently dysregulated pathways in human cancers. In NSCLC, the PI3K pathway has been implicated in tumorigenesis, disease progression, and resistance to cancer therapies (6, 7).

The PI3Ks are members of a family of heterodimeric lipid kinases, which are grouped into class I, II, and III isoforms (8, 9). Class IA subgroup of PI3Ks consist of a p110 catalytic subunit (p110α, *PIK3CA*; p110β, *PIK3CB*; p110δ, *PIK3CD*; and p110γ) and one of five p85-like regulatory subunits (p85α, p85β, or shorter forms). P110 alpha and beta are important in solid tumors, and delta and gamma, preferentially expressed in leukocytes, are important in hematological malignancies. The PI3K/AKT signaling network can be activated by receptor tyrosine kinases (RTKs), cytokine receptors, integrins, and G protein-coupled receptors (GPCRs). The PI3K pathway can be deregulated through a variety of mechanisms, including loss or inactivation of the tumor suppressor phosphatase and tensin homolog (PTEN), mutations or amplification of *PIK3* genes, as well as activation of tyrosine kinase growth factor receptors or oncogenes upstream of PI3K, e.g., EGFR and HER2.

Enormous efforts have been dedicated to the development of drugs targeting PI3K signaling for cancer therapy. More than 50 PI3K inhibitors have been designed and characterized, but only a minority have entered clinical trials (10).

MEN1611 is a PI3K inhibitor active on p110 alpha (both mutants and wild type), beta, and gamma isoforms. However, it is less potent on the beta (8.6-fold), gamma (2.2-fold), and delta (36-fold) isoforms than on the alpha isoform (11). By specifically targeting PI3KCA, this agent may be more efficacious and less toxic than pan-PI3K inhibitors; moreover, it has interesting features when compared with other PI3K inhibitors (12). Strong preclinical evidence (12) has supported its clinical development with the B-PRECISE-01, an ongoing phase Ib study to evaluate the safety and efficacy of MEN1611 plus trastuzumab with or without the estrogen receptor antagonist fulvestrant in patients with *PIK3CA*-mutated, HER2-positive, advanced, or metastatic breast cancer who have failed anti-HER2-based therapy (https://www.clinicaltrials.gov/ct2/show/NCT03767335). Moreover, a phase Ib/II study is currently ongoing to investigate MEN1611 in combination with cetuximab in patients with metastatic colorectal cancer (C-PRECISE-01) (https://clinicaltrials.gov/ct2/show/NCT04495621). This inhibitor has also been recently shown to inhibit the growth of allografts in nude immunodeficient mice of primary tumors derived from a high-frequency medulloblastoma murine model Ptch1+/−/Tis21KO medulloblastoma (13).

In this study, we investigate the efficacy of MEN1611 in models of NSCLC with a wild-type *PIK3CA* gene using *in vitro* studies on cancer cell lines and *in vivo* xenograft murine models.

Remarkably, a good efficacy of this inhibitor was observed in NSCLC devoid of *PIK3CA* gene mutations, but with constitutive activation of the PI3K/AKT pathway *in vitro* and *in vivo*. Moreover, by *in vitro* and *in vivo* studies, we show the synergistic effects of MEN1611 and EGFR-TKIs (erlotinib and gefitinib).

## Materials and methods

### Drugs

MEN1611 was synthesized at Menarini Ricerche (Pisa) and dissolved in dimethyl sulfoxide (DMSO) (Sigma-Aldrich, St. Louis, MO, USA; C5135) for *in vitro* studies and in DMSO (Sigma-Aldrich C5135)/Cremophor EL (Sigma-Aldrich D2650) solution (50%/50% v/v) and then formulated in 10% (w/v) hydroxypropyl-beta cyclodextrin (Sigma-Aldrich H107) and 10% (v/v) polyethylene Glycol 400 (Merck Millipore, Darmstadt, Germany) for the *in vivo* studies. Erlotinib Hydrochloride Salt and Gefitinib Free Base were from LC Laboratories (Woburn, MA, USA); they were dissolved for *in vitro* studies in DMSO, and gefitinib for the *in vivo* studies was formulated as for MEN1611.

### Reagents

The primary antibodies were as follows: EGFR (clone D09, kindly provided by O. Segatto) and phosphorylated-EGFR (Tyr1068_XP; #3777); Akt (#9272) and phospho-Akt (S473; #4060), S6 Ribosomal Protein (#2217) and phospho-S6 Ribosomal Protein (Ser235/236; #2211), eukaryotic translation initiation factor 4E (eIF4E)-binding protein 1 4EBP1 (#9452), extracellular signal-regulated kinase, Erk1/2 (p44/42 MAPK, #9102) and phospho-Erk1/2 (Thr202/Tyr204_XP; #4370), cleaved Caspase-3 (Asp175; #9661), PARP (#9542) (Cell Signaling Technology, Danvers, MA, USA), phospho-4EBP1 (62.Ser 65; sc-293124) (Santa Cruz, Dallas, TX, USA), and Ki67 (PA5-194629) (Thermo Fisher Scientific, Waltham, MA, USA), they were all used at a concentration of 1:1,000; anti-β Actin-Peroxidase (AC-15, A3854) (Sigma-Aldrich; 1:10,000), anti-α-tubulin (DM1A, T9026) (Sigma-Aldrich, 1:5,000), and anti-Glyceraldehyde-3-Phosphate Dehydrogenase, GAPDH (D16H11_XP, #5174) (Cell Signaling, 1:3,000) were used as loading control.

E-Cadherin (CD324) (AlexaFluoR647, 563571) (Becton Dickinson, Franklin Lakes, NJ, USA; 1:200), Phalloidin Conjugates (TRITC, P1951) (Sigma-Aldrich, 1:2,000), and Lectin Conjugates (FITC, L-0401) (Sigma-Aldrich, 1:1,000) were used for fluorescence. The secondary antibodies were as follows: goat anti-rabbit IgG (H+L)-HRP and goat anti-mouse IgG (H+L)-HRP were from Bio-Rad (Hercules, CA, USA); streptavidin Alexa Fluor-488 was from Life Technologies (Carlsbad, CA, USA). Biotin-labeled horse anti-rabbit Ig was from Vector Laboratories (Burlingame, CA, USA). Hoechst33342 was from Thermo Fisher Scientific. Harris Hematoxylin solution (HHS16), Eosin Y (318906) solution, Alcian Blue 8GX, MTT, 3-(4,5-methylthiazol-2-yl)-2,5-diphenyltetrazolium bromide, and Mowiol 4-88 were from Sigma-Aldrich. Fast Red was from Bio Optica Milano, SpA (Milan, Italy).

### Cell culture

The human breast cancer cell lines T-47D, MCF7, and MDA-MB-231 and NSCLC cell lines H1975, HCC827, HCC4006, and H460 from the American Type Culture Collection (ATCC; Manassas, VA, USA) were cultured in Roswell Park Memorial Institute (RPMI) 1640 medium (BioWhittaker, Lonza, EuroClone SpA, Milan, Italy) supplemented with 10 mM Hepes (pH 6.98–7.30), 1 mM l-glutamine, 100 U/ml of penicillin/streptomycin (BioWhittaker, Lonza), and heat-inactivated 10% fetal bovine serum (FBS) (Sigma-Aldrich). All cells were cultured at 37°C in a 5% CO_2_ humidified incubator. The EGFR-TKI-resistant cell lines HCC827-derived RA1, RA2, RB1, RB1.1, and RB2, established as previously described (4, 5), were cultured under the same experimental conditions.

### Formation and growth of spheroids

NSCLC spheroids were prepared using the “dome” formation protocol (Corning manufacturing instructions): briefly, 5 × 10^4^ single-cell resuspension was mixed 1:10 with chilled Matrigel, and 50 µl of single domes in 24-well plates was placed upside down in the incubator at 37°C for 10 min. Complete RPMI medium was added carefully to each well, and the spheroids were left to grow until they reached a diameter of 300 µm for approximately 10–14 days.

### Cell viability assays and drug combination analysis

The MTT method was employed as previously described to determine the viability of cancer cell lines (5). Briefly, MTT stock solution (5 mg/ml in H_2_O, sterilized by filtration) was stored at 4°C for 1 month. Cells were seeded into 96-well plates at a density of 10–20,000 cells/well. After overnight incubation, cells were exposed to selected inhibitors at the indicated concentrations for 72 h; then, they were gently washed, incubated with MTT for 4 h, and processed for color detection with DMSO. The resulting purple solution was spectrophotometrically measured at 570 nm using a Varioskan Lux instrument and Skanit software (Thermo Fisher Scientific). The optical density values were expressed as a percentage of cell survival and normalized with the value of cells treated with vehicle (DMSO), and the data were analyzed using GraphPad Prism v6.0c software (San Diego, CA, USA).

The synergy between erlotinib/gefitinib and MEN1611 was evaluated using the Chou–Talalay method (14) (http://www.combosyn.com) as previously described (5). In brief, the cells were treated with gefitinib and MEN1611 (approximately 6–8 concentrations, range 0.016–10 μM) at a ratio of 1:1. Each drug was also used alone at the same concentrations. Cell survival was determined using MTT assays. Each data point was analyzed in triplicate. CompuSyn software (ComboSyn Inc., Paramus, NJ, USA) was used to determine the dose–effect curves for single and combination treatments. Next, non-linear regression trend lines were used to calculate the combination index (CI) values and to assess the nature of drug interactions that can be additive (CI = 1), antagonistic (CI > 1), or synergistic (CI < 1).

### Apoptosis and cell cycle analysis

Upon treatment, cancer cell lines were collected and stained with propidium iodide at 50 µg/ml in 0.1% Triton X-100/0.1% sodium citrate in phosphate-buffered saline (PBS) for 30 min at 4°C. Cell cycle and apoptosis analyses were performed at FACSCalibur (Becton Dickinson, Milan, Italy) and quantified in linear and logarithmic histogram plots, respectively.

### Preparation and Western blotting analysis of protein extracts

Cells were washed twice in PBS and then lysed in a buffer containing 50 mM Tris-Cl (pH 7.6), 150 mM NaCl, 2 mM EDTA, 1% NP-40, 0.25% sodium deoxycholate, 1× Phosphatase Inhibitor Cocktails 2 and 3 (Sigma-Aldrich, Italy), and 1× Protease Inhibitor Cocktail (P8340 Sigma-Aldrich). Proteins (25–50 μg) in cell lysates, quantified with the Bio-Rad DC Protein Assay kit, were then separated by 8%–15% sodium dodecyl sulfate–polyacrylamide gel electrophoresis (SDS-PAGE) and transferred to nitrocellulose membranes, 0.45 μm (GE Healthcare Life Sciences, Milwaukee, WI, USA). The membranes were blocked with 5% non-fat milk (EuroClone SpA) or 2% bovine serum albumin (BSA) (Sigma-Aldrich) in 1× Tris-buffered saline (TBS) pH 7.6–8.0 containing 0.1% or 0.2% Tween 20 (TBST) for 2 h at room temperature (RT) and subsequently probed with primary antibodies in 5% non-fat milk or 2%–5% BSA in TBST overnight at 4°C. Then, membranes were washed with TBST and probed with horseradish peroxidase-conjugated secondary antibodies in 5% non-fat milk in TBST for 1 h at RT. ChemiDoc XRS Bio-Rad was used for image acquisition with a chemiluminescent camera, and signals were quantified using ImageLab 4.0 Bio-Rad software.

### *In vivo* NSCLC murine model xenograft studies

All animal procedures were performed in accordance with the current guidelines of the European Ethical Committee (directive 2010/63/EU) and were in strict compliance with the protocols approved by the Italian Ministry of Health (Authorization Nos. 872/2015-PR and 898/2018-PR). All efforts were made to minimize animal pain and discomfort. NOD scid gamma mouse (NSG) mice (4- to 6-week-old) were subcutaneously implanted into their right flank with 5 × 10^6^ NSCLC cells in 200 μl of PBS. Once the tumors became palpable, the mice were monitored twice a week, and the size of the nodules was measured using a digital caliper. Each tumor volume (TV) was calculated using the ellipsoid formula, TV = (L × W2)/2, where L is the length of the nodule and W is the width. When the tumors reached a volume of approximately 150 to 200 mm^3^, the mice were randomized into experimental and control groups (n = 5 and 6 in each group), and treatment was started. The mice in the control group were treated with vehicle solution DMSO/Cremophor EL (50%/50% v/v), diluted 10 times in a solution containing 10% 2-hydroxypropyl-β-cyclodextrin and 10% Polyethylene Glycol 400 in distilled water. Mice in the experimental group were treated with MEN1611 (6.5 mg/kg/day) or gefitinib (50 mg/kg/day) or with both drugs by two independent oral gavages. The inhibitors were dissolved in a DMSO/Cremophor EL mixed solution and prepared in a 10-fold diluted solution. Mice were treated for 12–20 consecutive days by oral gavage. At the end of treatment, all mice were euthanized with intraperitoneal injections of tiletamine/zolazepam (800 mg/kg) and xylazine (100 mg/kg), and tumors were excised, measured, photographed, and pathologically examined. Tumors were divided into two parts: one part was flash frozen in liquid nitrogen for molecular analysis, and the other was fixed in 4% paraformaldehyde (PFA) in PBS by overnight immersion for immunohistochemical analysis. For protein phosphorylation studies, tumors were harvested 4 h after the last dose of drug gavage.

### Immunohistochemistry

Tissue samples were fixed in 4% formalin, dehydrated, embedded in paraffin, and cut into 8-µm sections. Slides with paraffin sections were boiled for 20 min in antigen retrieval sodium citrate buffer (10 mM, pH 6.0) and allowed to cool at room temperature. Then, they were incubated with a blocking buffer (0.1% Triton X-100, 1% BSA, and 4% normal donkey serum in 1× PBS) for 1 h and then incubated overnight at 4°C with primary antibodies diluted in the blocking buffer. Immunostained positive cells were detected by fluorescence-conjugated secondary antibodies and counted in representative tumor areas; the percentage of positive cells was calculated with respect to nuclei.

### Hematoxylin and eosin and Alcian Blue Fast Red staining

Paraffin sections from NSCLC xenografts were washed with xylene and rehydrated with a decreasing concentration of ethanol. For hematoxylin and eosin (H&E) staining, samples were washed and stained with Hematoxylin Harris solution, washed in acidic alcohol, and cleared in Bluing solution. After tap water washing, samples were stained with eosin Y solution and then dehydrated with absolute ethanol and xylene. Eukitt was used as the mounting medium. For Alcian Blue Fast Red staining, after washing with distilled water, samples were stained with Alcian Blue pH 2.5 solution, washed in water, and counterstained with neutral red stain (Fast Red). Upon dehydration in absolute alcohol, they were mounted using Eukitt. Images were recorded using an Olympus BX41 microscope equipped with an Olympus SP-350 camera.

### Immunofluorescence

Cells, seeded on glasses coated with poly-l-lysine (100 µg/ml in H_2_O) or on 35-mm tissue culture plastic plates, were fixed with 3.7% paraformaldehyde in PBS. Cells permeabilized with 0.2% Triton X-100 in PBS for 6 min at room temperature and treated with blocking solution (1× PBS, 1% BSA) were incubated with primary antibodies, followed by Alexa conjugated secondary antibodies. Nuclei were stained with Hoechst 33258 (1 μg/ml). Coverslips were finally mounted with Mowiol 4-88 mounting media and viewed on an Olympus AX70 or BX53F2 fluorescence microscope supported by an Olympus XM10 camera and Olympus CellSens Standard 1.8.1 software or an Olympus FV1200 confocal laser scanning microscope using an UPlanFLN 40× immersion oil objective (NA = 1.30) with an optical pinhole at 1 AU and a multiline argon laser at 488 nm, HeNe ion laser at 543 nm, and blue diode laser at 405 nm as excitation sources. Samples were also analyzed with a 20× air or 40× air. Confocal images were processed using ImageJ or Fiji and Adobe Photoshop.

### Spheroid treatment and staining

Spheroids were treated with a vehicle or inhibitor in a complete tissue culture medium at a concentration of 1–5 µM for 48 h. Next, cultured spheroids were gently washed twice in PBS and fixed with 4% paraformaldehyde for 1–2 h at room temperature on a rotation wheel. The spheroids settled at the bottom of the tube by gravity were washed in PBS and permeabilized in PBS supplemented with 3% BSA plus 0.5% Triton X-100 for 4 h at +4°C. Primary antibodies were added and incubated at 4°C overnight. Nuclei were counterstained with Hoechst 33342 (1 µg/ml) in PBS for 30 min. Samples were imaged using a confocal microscope in the µ-Plate 96 Well Black (Ibidi n. 89626).

### Image analysis

Images were acquired using an Olympus BX53F or BX53F2 inverted fluorescence microscope. The following were analyzed: 4–10 images/slides, 2–4 slices/nodules, and 2–3 nodules/condition. At least 20,000 nuclei were counted for each experimental condition of the RA1, RB1, and RA2 cell lines, and ~5,000 and ~12,000 nuclei were counted for the HCC827 cell line treated with gefitinib and MEN1611, respectively. RGB images were analyzed using Fiji v2.1 (15) to automatically calculate the percentages of Ki67+ cells. For each RGB image, the Weka trainable classifier (16) was used to segment the blue channel image to distinguish the background and murine nuclei from the human nuclei. The human nuclei were then filled (“Fill holes method”), separated (“‘Watershed method”), and counted using the “Analyze particles” tool, using 50 or 200 pixels^2^ as the minimum size for images acquired at BX53F and BX53F2. As regards the count of Ki67+ cells, the green channel image was binarized using a threshold of 30–50, filled (“Fill holes method”), and multiplied by the segmented image of human nuclei (mentioned above). Finally, the Ki67+ human cell counts were obtained using the “Analyze particles”. Less straightforward images were manually counted to distinguish the background and murine nuclei from those of human.

The peculiar vacuolar structures observed in tissue samples of nodules treated with MEN1611 and stained with H&E were analyzed using the open-source software Ilastik using the workflow (17). Briefly, selection and label assignment of the classes of interest (Tissue and Lumen) through “Pixel classification + Object classification” was followed by a threshold setting using the simple method, configuring the sigmas for smoothing the probability image and setting a size filter with a minimum size of 100 pixels^2^. Based on the features of the labeled regions and setting a threshold of the software learned to predict the segmentation and in batch processing showed the predictions as an overlay for all the images of interest. The predictions were then exported and used for post-processing in ImageJ, where lumens were quantified and analyzed by size.

### Statistical analysis

Data and statistical analysis were performed using GraphPad Prism version 7.0 software (GraphPad Software, San Diego, CA, USA) and R v4.1.0 (18). Gaussian distribution and homoscedasticity of datasets were tested by the Kolmogorov–Smirnov test and Bartlett’s test, respectively. Differences in groups were compared using t-test, Welch’s t-test, one-way ANOVA, or pairwise Wilcoxon Rank Sum test, as appropriate. Correction for multiple comparison testing was performed by Dunnett’s method or the Benjamini–Hochberg method, as appropriate. A p-value <0.05 was considered statistically significant. Data are represented as mean ± standard deviation, except for tumor growth curves where the standard error was used as a measure of sample dispersion. Statistical methods for each experiment are provided in the figure legends.

## Results

### Sensitivity of NSCLC cell lines with mutated or wild-type *PIK3CA* gene to the PI3K inhibitor

**Figure 1:**
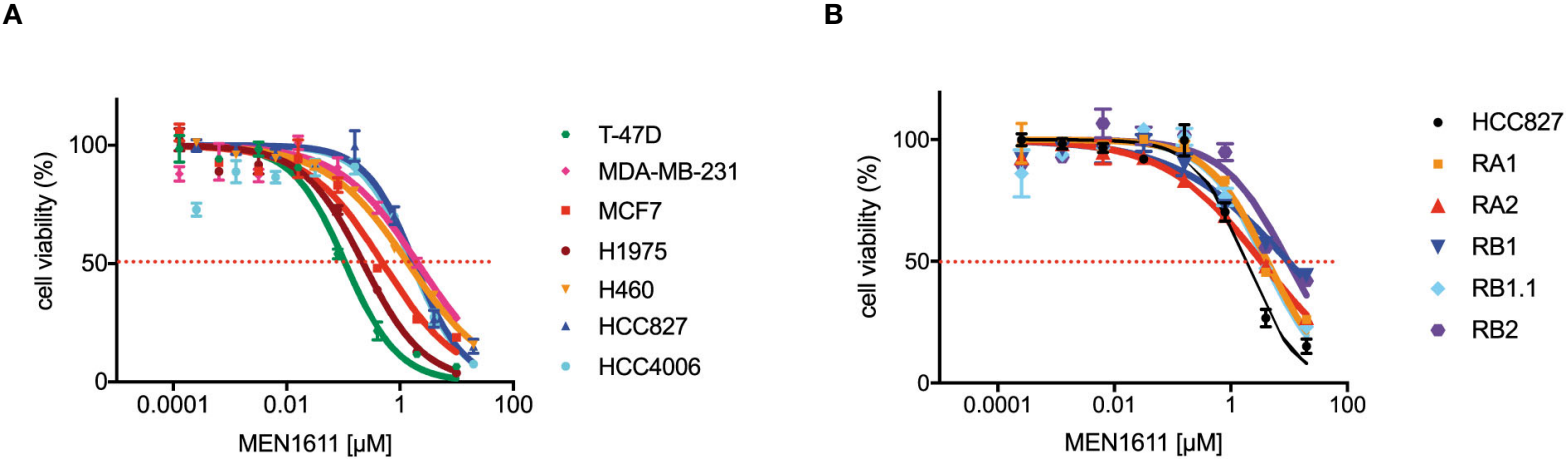
Effects on MEN1611 on cancer cell viability. Representative MEN1611 dose–response curves of cancer cell lines: **(A)** T-47D, MDA-MB-231, and MCF7 (breast cancer cell lines) and H1975, H460, HCC827, and HCC4006 (NSCLC cell lines); **(B)** EGFR-TKI-sensitive cell line HCC827 and HCC827-derived-resistant cell lines RA1, RA2, RB1, RB1.1, and RB2.

To assess the potential impact of MEN1611 on the viability of NSCLC cell lines sensitive and resistant to EGFR-TKIs (i.e., erlotinib and gefitinib), dose–response experiments were conducted on NSCLC cell lines with a wild-type or mutated *PIK3CA* gene (**Figure 1A**). MEN1611 decreases the viability of all NSCLC cell lines with an estimated IC_50_ in the low micromolar range (**Figure 1A**; **Table 1**). Breast cancer cell lines with a wild-type or mutated *PIK3CA* gene were also assayed, and the IC_50_ values, in the low micromolar range, were similar to those previously reported for the CH5132799 drug (PA-799) (19). Some breast cancer cell lines, such as T-47D and MCF7, were slightly more sensitive than the NSCLC cell lines to the PI3K inhibitor, likely due to mutations in the kinase and helical domains (**Figure 1A**; **Table 1**). Among the NSCLC cell lines, HCC827, which is sensitive to EGFR-TKIs, showed a higher sensitivity to MEN1611 when compared to its erlotinib-resistant derived cell lines. The differences observed were all statistically significant except for RB2, which was likely due to its higher intra-assay variability (**Figure 1B**; **Table 2**).

**Table 1:** Breast and NSCLC cancer cell lines, gene mutations, and sensitivity to MEN1611.

Cell line	PI3KCA	EGFR	MEN1611 IC_50_ (µM)
**T-47D**	H1047R	wt	0.08 ± 0.02
**H1975 ^†^**	G118D	L858R; T790M	0.41 ± 0.14
**MCF7**	E545K	wt	1.37 ± 0.55
**HCC4006 ^†^**	wt	ΔL747-E749; A750P	1.51 ± 0.27
**HCC827 ^†^**	wt	ΔE746-A750	1.74 ± 0.35
**H460 ^†^**	E545K	wt	1.92 ± 0.63
**MDA-MB-231**	wt	wt	2.15 ± 0.33

EGFR and PI3KCA mutations are shown. †: lung cancer cell lines. MEN1611 IC_50_ (μM) mean values +/- SEM are representative of three independent experiments. In each experiment R square was > 0.9. Wild type:wt.

**Table 2:** Sensitivity to MEN1611 of NSCLC with wild-type PI3KCA.

Cell line	IC_50_ MEN1611 (µM)
**HCC827**	1.74 ± 0.35
**RA1**	4.17 ± 0.49*
**RA2**	4.98 ± 0.48**
**RB1**	6.63 ± 0.53**
**RB1.1**	3.59 ± 0.16*
**RB2**	6.60 ± 1.92

MEN1611 IC_50_ (µM) mean values ± SEM are representative of three independent experiments. In each experiment, R^2^ was >0.9. Unpaired t-test was performed to evaluate differences in sample means.

NSCLC, non-small cell lung cancer.

*p < 0.05, **p < 0.01.

**Figure 2:**
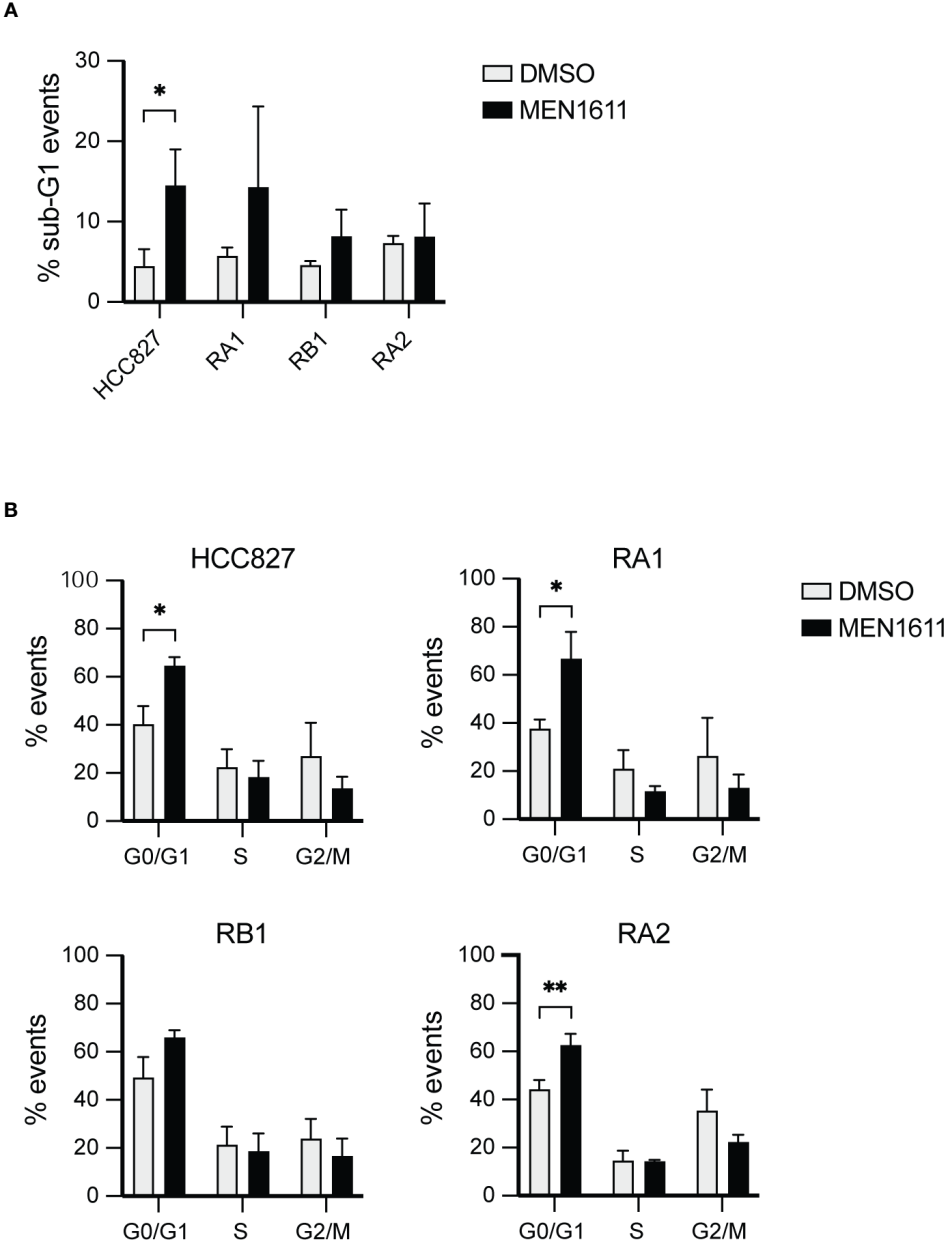
Flow cytometry analysis by DNA content measurement of cell cycle and apoptosis. **(A)** The histograms represent the percentage of events recorded in the cancer cell lines indicated after treatment with DMSO or MEN1611 (5 µM) for 48 h. **(A)** % sub-G1 events apoptotic cells; **(B)** % G0/G1, S, G2/M cell cycle stages events. The values shown are representative of three independent experiments. Unpaired t-test was performed to evaluate differences in sample means. *p < 0.05, **p < 0.01. DMSO, dimethyl sulfoxide.

Apoptosis induced by MEN1611 treatment in the NSCLC was modest, and less than 20% of the cell population had hypodiploid sub-G0/1 apoptotic peak at 48 h after drug treatment, as assessed by propidium iodide staining and cytofluorimetric analysis (**Figure 2A**; **Table S1**).

**Figure 3:**
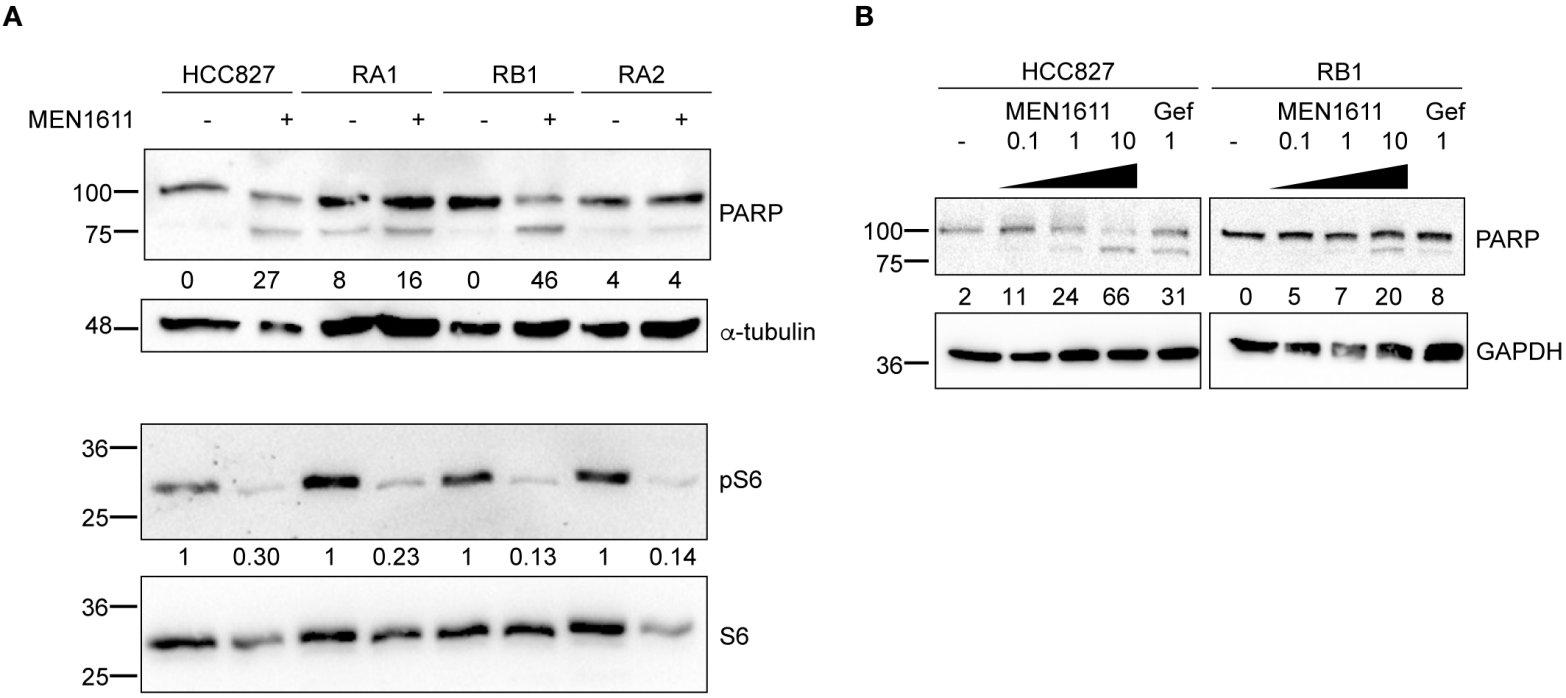
Effects of MEN1611 on apoptosis. Western blotting analysis of cleaved PARP (cPARP) in cell lysates of MEN1611-treated (5 µM) NSCLC cell lines. **(A)** Quantification of cPARP (lower band in the doublet) is shown as a percentage of total PARP protein. Alpha-tubulin was used as loading control. Decrease in S6 phosphorylation (pS6) signals shows efficacy of MEN1611. Quantification of pS6 relative to the total S6 protein was performed. The pS6/S6 values of MEN1611-treated samples were normalized to those of the DMSO for each cell line. **(B)** Western blotting analysis of cPARP in lysates of HCC827 and RB1 cell lines treated with increasing concentrations of MEN1611 (0.1 µM, 1 µM, and 10 µM) or gefitinib (1 µM) for 48 (h) Quantification of cPARP (lower band in the doublet) is indicated as a percentage of total PARP protein. GAPDH was used as loading control. Gef, gefitinib.

Apoptosis was also investigated by poly(ADP-ribose) polymerase cleavage (cPARP) in cell lysates of erlotinib-sensitive or erlotinib-resistant cell lines treated with MEN1611 (at 5 µM concentration) for 48 h. Inhibition of S6 phosphorylation was assessed in cell lysates by Western blotting to ensure the efficacy of the PI3K inhibitor under our setting conditions. Samples treated with MEN1611 showed increased PARP cleavage in all cell lines, except for RA2, as shown by band signal quantification of cPARP with respect to the full-length PARP (**Figure 3A**). Moreover, the signal of cPARP correlates with the concentrations of MEN1611 (0.1–10 µM) used for treating the EGFR-TKI-sensitive (HCC827) and erlotinib/gefitinib-resistant cell line, RB1 (**Figure 3B**). Cytofluorimetric analysis also showed that after exposure to MEN1611 (5 µM) for 48 h, the percentage of cells in the G0/G1 phase increased, while the percentage of cells in the S and/or G2/M phases decreased (**Figure 2B**; **Table S1**), similar to the G1 cell cycle arrest.

### Modulation of the constitutively activated PI3K/AKT/mTOR pathway in cancer cell lines harboring wild-type or mutated *PIK3CA* gene by MEN1611

**Figure 4:**
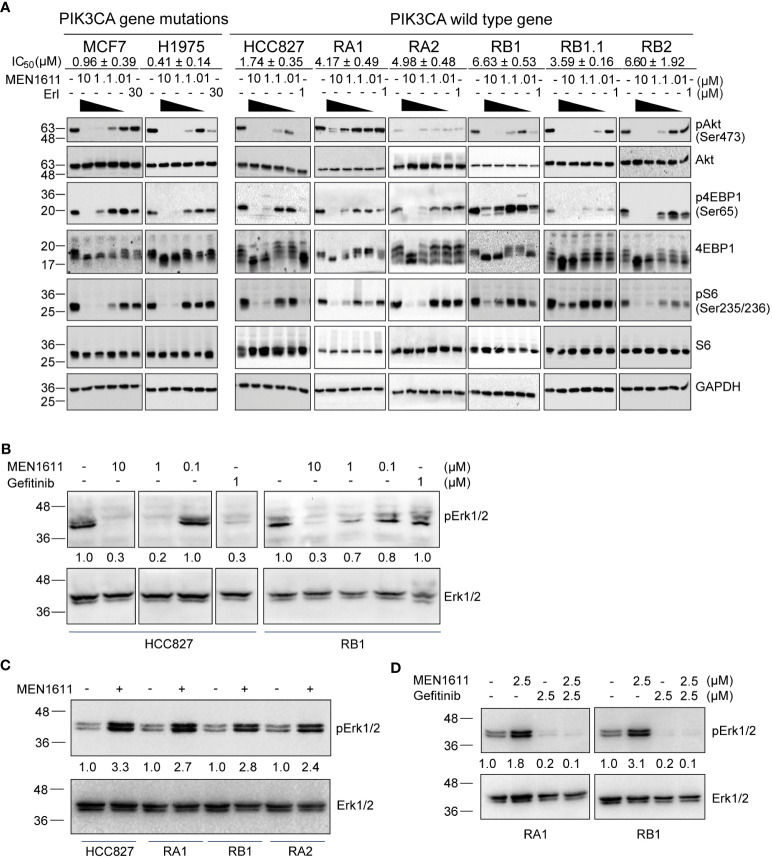
Effects of MEN1611 on signaling pathways. **(A)** Western blotting analyses of PI3K signaling pathways in lysates of PI3KCA mutated cell lines (MCF7 and H1975) and cell lines with wild-type PI3KCA, EGFR-TKI-sensitive (HCC827) and EGFR-TKI-resistant NSCLC cell lines (RA1, RA2, RB1, RB1.1, and RB2) treated with MEN1611 (10 to 0.01 µM) or erlotinib (30 or 1 µM) for 2 (h) IC_50_ mean values ± SEM are reported. pAkt (Ser473), Akt, p4EBP1(Ser65), 4EBP1, pS6(Ser235-236), and S6 signals are shown. GAPDH was used as loading control. **(B)** Western blotting analyses of Erk/pErk in cell lysates of NSCLC cell lines: HCC827 and RB1 treated with MEN1611 (10 to 0.1 µM dose concentrations) or gefitinib (1 µM) for 2 h **(C)** HCC827, RA1, RB1, and RA2 treated with MEN1611 (5uM for 48h μM) for 48 h **(D)** RA1 and RB1 cell lines treated for 48 h with a combination of MEN1611 and gefitinib (1:1) at the indicated drug concentrations for 48 h. Quantification of pERK1/2 relative to the total ERK1/2 protein was performed. The pERK1/2/ERK1/2 values of treated samples were normalized to those of the DMSO for each cell line in **(C, D)**.

The PI3K/Akt/mTOR pathway is considered one of the most attractive targets for the development of anticancer agents, and it is constitutively activated in several cancer cells including NSCLC. Therefore, to explore the impact of MEN1611 treatment on this pathway in NSCLC with a wild-type *PIK3CA* gene, the phosphorylation status of known PI3K downstream targets was examined. A breast cancer cell line (MCF7) and a NSCLC cell line (H1975) with a mutated *PIK3CA* gene were also included in the experiments. Lysates of cells treated with vehicle (DMSO) or increasing concentrations of MEN1611 (0.01 µM, 0.1 µM, 1 µM, and 10 µM) or erlotinib (1 µM or 30 µM) were analyzed by SDS-PAGE and Western blotting. Upon treatment with the PI3K inhibitor, decreased levels of pAKT (Ser473), p4EBP1 (Ser65), and pS6 (Ser235/236) were observed in all cell lines with either a wild-type or mutated *PIK3CA* gene. Remarkably, the inhibitor was effective on the pathway in both erlotinib/gefitinib-sensitive and erlotinib/gefitinib-resistant NSCLC cell lines (**Figure 4A**).

It has been previously reported that PI3K regulates ERK signaling in many tumor models (20–22). Therefore, we investigated the status of ERK1/2 in the lysates of HCC827 and RB1 cell lines treated with vehicle or MEN1611. At 2 h post-treatment, MEN1611 inhibited ERK1/2 phosphorylation at both 10 and 1 µM concentrations as also shown by band signal quantification of pERK1/2 relative to total ERK protein expression levels (**Figure 4B**). In contrast, at 48 h post-treatment, the levels of pERK1/2 in MEN1611-treated samples (5 µM) were higher than those in vehicle-treated samples (**Figure 4C**), suggesting overactivation of a compensatory pathway. Moreover, pERK1/2 was downregulated in EGFR-TKI-resistant cells, RA1 and RB1, treated with gefitinib or with the combination of MEN1611 and gefitinib, suggesting that drug combination could be beneficial to overcome ERK signaling overactivation (**Figure 4D**).

### Synergistic effect of EGR-tyrosine kinase inhibitors and MEN1611 in EGFR-TKI-resistant NSCLC cell lines

Targeting both EGFR and PI3K signaling pathways may be useful for optimal therapeutic activity in cancer to prevent or overcome EGFR-tyrosine kinase resistance and/or activation of compensatory pathways. Therefore, studies were performed to determine the effect of PI3K and EGFR inhibitors on selected HCC827-derived cell lines, RA1, RA2, and RB1, 72 h after treatment. The EGFR-TKIs erlotinib and gefitinib induce similar effects *in vitro* in both MTT assays and biochemical studies (5). Indeed, cellular extracts of HCC827 and the derived-resistant RA1, RB1, and RA2 cells show a decrease in EGFR phosphorylation upon treatment with both EGFR inhibitors (**Figure S1**). Therefore, the following experiments were performed using gefitinib.

MEN1611 (20–0.016 µM) and gefitinib (20–0.016 µM) were used alone or in combination at a 1:1 ratio. The Dm data for the single drug and their combination and the CI are summarized in **Table 3**.

**Table 3:** Synergy between MEN1611 and gefitinib in NSCLC cell lines.

	Drug	RA1	RA2	RB1
**Dm (µM)**	Gef	> 10	> 10	> 10
	MEN1611	4.9 –7.2	2.5 –5.9	5.5 – 10.0
	Gef+MEN1611	1.1 –1.9	1.9 –4.9	3.5 – 6.1
**CI**	Gef+MEN1611	0.1 –0.2	0.2 –0.4	0.2 – 0.4

Median-effect dose (Dm) of single and combination drugs and relative combination index (CI) of EGFR-TKi resistant NSCLC cell lines, calculated at 50% of fraction affected. Gefitinib, Gef.

Using the survival data generated from the MTT assays, dose–effect, CI, and isobologram plots were generated for the drug pair with CompuSyn software; the fold changes in fraction affected due to the drug combination and the dose reduction index (DRI) of MEN1611 are also tabulated (**Figure S2**). These values, in addition to CI values lower than 1, indicate that MEN1611 and gefitinib are synergistic in the RA1, RB1, and RA2 EGFR-TKI-resistant NSCLC cell lines (**Table 3**; **Figure S2**).

### Therapeutic response to MEN1611 alone and in combination with gefitinib in NSCLC xenograft murine models

**Figure 5:**
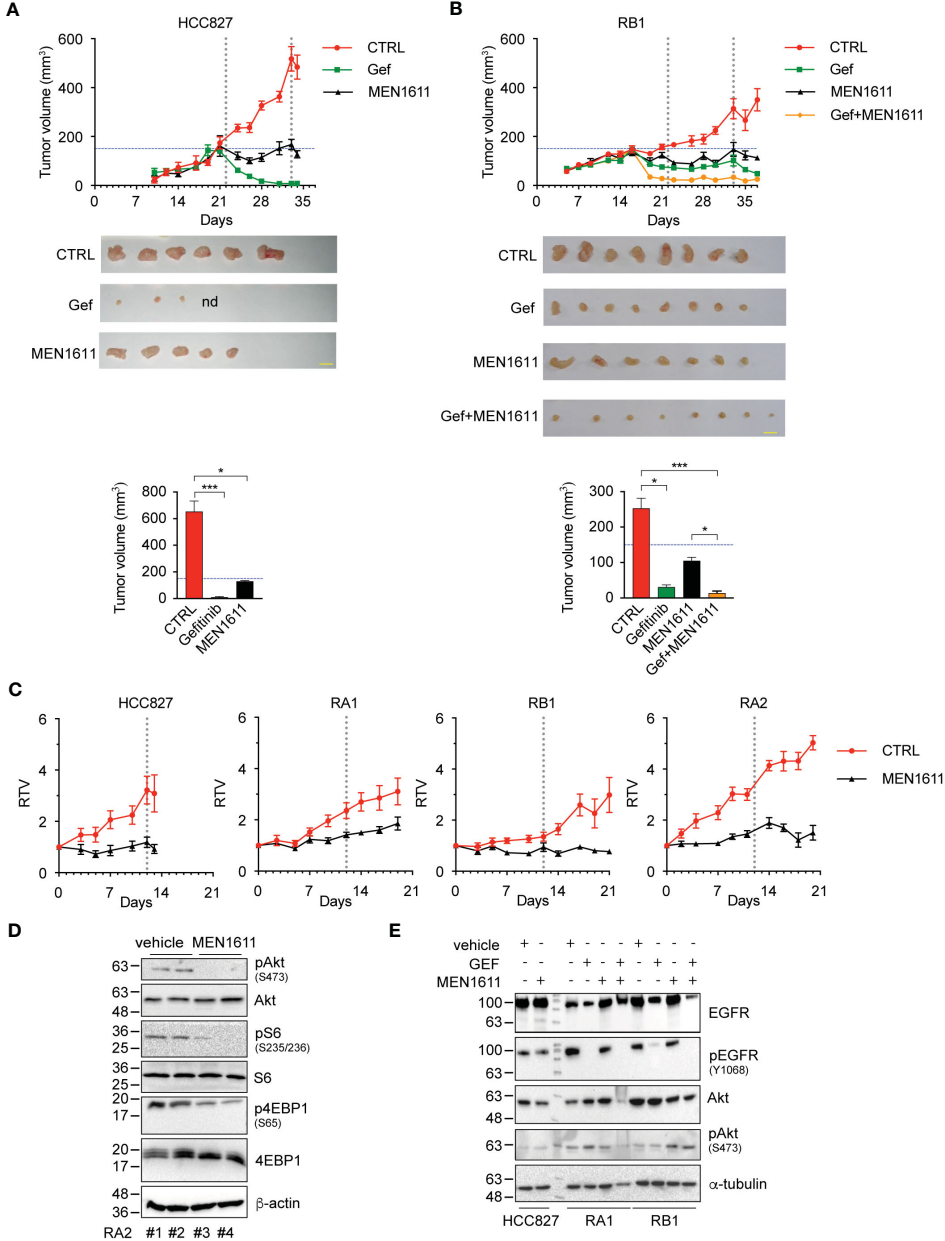
*In vivo* studies of drug efficacy in HCC827 and RB1 xenograft murine models. **(A)** Tumor growth curves in NSG mice (n = 4–6), subcutaneously injected in the flank with HCC827 and RB1 cell lines and orally treated with vehicle (CTRL) (red), gefitinib at 50 mg/kg (green), MEN1611 at 6.5 mg/kg (black), and gefitinib plus MEN1611 (yellow) for 12–18 days. Mice were treated with drugs when the tumor volumes were 150 mm^3^ approximately. Data are shown as mean ± standard error. Pairwise Wilcoxon rank sum tests with Benjamini–Hochberg correction for multiple testing were used to compare the experimental groups at every time point. HCC827: MEN1611 and gefitinib vs. CTRL, p < 0.05 at day 24 and from day 31 to 34; p < 0.01 at days 26 and 28. RB1: MEN1611 vs. CTRL, p < 0.05 at days 35 and 37; p < 0.01 at day 28; p < 0.001 at days 23, 26 and 30; gefitinib vs. CTRL: p < 0.05 from day 33 to 37; p < 0.01 at day 19; p < 0.001 from day 21 to 30; gefitinib/MEN1611 treatment vs. CTRL, MEN1611 or gefitinib: p < 0.05 from day 33 to 37; p < 0.001 from day 19 to 30. p-Values not listed above are not significant. Images of xenograft excised nodes (scale bars, 1 cm) and histograms of the tumor volumes post-treatment of HCC827 **(A)** and RB1 cells **(B)** are shown. nd indicates not detected. Pairwise comparison of experimental groups was performed by Kruskal–Wallis test with Benjamini–Hochberg correction for multiple testing. *p < 0.05, ***p < 0.001. p-Values not listed above are not significant. Exact p-values are listed in **Table S2**. **(C)** Tumor growth curves of HCC827, RA1, RA2, and RB1 xenografts. Relative tumor volume of xenograft growth in NSG mice (n = 6) treated with vehicle (CTRL) (red) or MEN1611 at 6.5 mg/kg (black) for 12 days (HCC827) or 18–20 days (RA1, RA2, and RB1). Mice were treated with drugs when the tumor volumes were approximately 120–150 mm^3^ (T0). Data are shown as mean ± standard error. Pairwise Wilcoxon rank sum test with Benjamini–Hochberg correction for multiple testing was used to compare the experimental groups at each time point. HCC827: MEN1611 vs. CTRL, p < 0.05 at days 5, 7 and 12; p < 0.01 at days 10 and 13. RA1: MEN1611 vs. CTRL, p < 0.01 from day 9 to 14. RA2: MEN1611 vs. CTRL: p < 0.01 from day 3 to 18; p < 0.05 at day 20. RB1: MEN1611 vs. CTRL, p < 0.05 at day 7, and from day 17 to 21; p < 0.01 at days 10 and 14. p-Values not listed above are not significant. Exact p-values are listed in **Table S1**. MEN1611 treatment reversibly impacts the PI3K/AKT pathway *in vivo* in xenograft murine models. **(D)** Western blotting analysis of total protein lysates of RA2 nodules obtained from mice treated with vehicle (#1 and #2) or MEN1611 (#3 and #4). Blots probed with Akt/pAkt(Ser473), 4EBP1/p4EBP1(Ser65), and S6/pS6(Ser235-236) specific antibodies are shown. Beta-actin was used as loading control. **(E)** Western blotting analysis of total extracts of HCC827, RA1, and RB1 xenograft nodules from mice treated with gefitinib, MEN1611, and gefitinib/MEN1611. Blots probed with Akt/pAkt(Ser473) and EGFr/pEGFR specific antibodies are shown. Alpha-tubulin was used as loading control.

To further evaluate the efficacy of the PI3K inhibitor and combined gefitinib and MEN1611 treatment *in vivo*, we generated xenograft mouse models with HCC827, RA1, RB1, and RA2 NSCLC cells as described in the Materials and Methods. After inoculation of cancer cells in the flanks of NSG mice, tumor growth was monitored over time using a caliper. In the experiments, the mice were treated with a vehicle, MEN1611 (HCC827, RA1, RB1, and RA2) (**Figures 5A, B**), gefitinib (HCC827, RB1) (**Figures 5A, B**), or a drug combination (RB1) (**Figure 5B**). Reduced growth of tumors or a cytostatic effect was observed during treatment with MEN1611 in all xenografts; statistical analysis of the node size differences is indicated in the caption (**Figures 5A, B**) and in **Table S2**. In the RB1 xenograft mice, MEN1611 was also assayed in combination with gefitinib (**Figure 5B**). RB1 is an HCC827-derived cell line resistant to EGFR inhibitors (erlotinib and gefitinib) in 2D cell culture. The cell line has a stable morphological phenotype, and response to TKI and shows stable molecular signatures in the early *in vitro* passages (up to 12 passages) (4, 5). As expected, the growth of the gefitinib-sensitive HCC827 xenograft tumor was almost completely abrogated (**Figure 5A**). Regarding the RB1 xenograft, we expected greater growth in the group of mice receiving gefitinib. The RB1 cancer cell line was resistant *in vitro* in 2D culture with an IC_50_ of more than 10 µM; however, daily treatment with gefitinib and possibly a selection of cells in the implantation phase or a contribution of the microenvironment made the RB1 cells *in vivo* partially responsive to gefitinib (**Figure 5B**). Nevertheless, the RB1 xenograft mouse group treated with a combination of both inhibitors showed tumor volumes smaller than those of mice treated with a single drug. Statistically significant differences observed during the treatment are described in the caption (**Figure 5**) and in **Table S2**.

Remarkably, the synergistic effect of MEN1611 and gefitinib is consistent with the *in vitro* cell experiments (**Table 3**; **Figure S2**). Importantly, the mice did not show any significant weight loss when compared with the control group, suggesting that the monotherapy and combination therapy were both well tolerated (**Table S3**).

To evaluate the mechanism of action of MEN1611 *in vivo*, immunoblotting studies were performed on nodule tissues obtained from mouse xenografts. A decrease in pAkt, pS6, and p4EBP1 signals in the RA2 tumor nodule extracts 4 h after the last oral drug administration by gavage was observed (**Figure 5D**), proving that MEN1611 suppresses tumor growth in the xenograft model through inhibition of the PI3K/AKT pathway. However, the timing of biochemical studies is important. In extracts of nodes examined at 24 h post-treatment, no variation in pAkt was detected in HCC827, RA1, and RB1 nodules, suggesting that the effect of MEN1611 is reversible and transient (**Figure 5E**). Washing-out experiments *in vitro* confirmed this hypothesis; indeed, in HCC827 and RB1 cell lysates, pAkt(S473), pS6(Ser235/236), and p4EBP1(Ser65) phosphorylation signals in drug wash-out samples were similar to those in the untreated samples (**Figure S3**).

Cell proliferation (Ki67) and apoptosis (cleaved Caspase-3) markers were used in immunochemistry studies in xenograft nodules. No significant differences were observed in the number of Ki67+ cells in node sections of mice treated with MEN1611, except for HCC827, where the number of Ki67+ cells slightly decreased (**Table 4A**). Instead, a statistically significant increase in Ki67+ cells was detected in the RB1 nodules of mice treated with the drug combination, suggesting that the residual tumor cells were actively proliferating. A small but statistically significant increase of cleaved Caspase-3 was observed in HCC827, RA1, and RA2 xenografts (**Table 4B**). Representative images of Ki67 and cleaved Caspase-3 immunostaining are reported in **Figure S4**.

**Table 4:** Immunohistochemistry analysis of xenograft nodule sections.

(A) % Ki67^+^ cells/nuclei				
Drug	HCC827	RA1	RB1	RA2
Vehicle	32.6 ± 3.2	28.1 ± 4.2	25.4 ± 3.2	33.9 ± 3.2
Gefitinib	25.3 ± 6.5*	n.d.	32.6 ± 8.9	n.d.
MEN1611	24.7 ± 5.8*	29.6 ± 8.4	28.5 ± 6.5	30.4 ± 5.5
Gefitinib+MEN1611	n.d.	n.d.	37.1 ± 3.9**	n.d.

**Table:** 

(B) % cleaved Casp3/nuclei				
Drug	HCC827	RA1	RB1	RA2
Vehicle	5.8 ± 1.4	8.3 ± 1.2	9.8 ± 2.1	10.9 ± 2.6
MEN1611	8.4 ± 1.5*	12.2 ± 2.3**	11.4 ± 1.2	13.7 ± 1.3*

(A) Counts of Ki67+ cells in xenograft nodules. Images were captured from 4–6 slides and 2–4 nodules; at least 24 fields and 3,000 nuclei for each experimental condition were analyzed. Statistical analysis was achieved by ANOVA with Dunnett’s multiple comparison test (HCC827, RB1) or t-test (RA1, RA2). Geftinib, Gef; n.d., not determined. (B) Counts of cleaved Caspase-3-positive cells in xenograft nodules. Images were captured from 3–6 slides and 1–2 nodules; at least 5–9 fields and 500–1,500 nuclei for each experimental condition were analyzed. Statistical analysis was performed by unpaired t-test. Data are shown as mean ± standard deviation. Asterisks refer to pairwise comparison of treated samples vs. vehicle group. * p < 0.05; ** p < 0.01; not significant p-values are not shown.

### Tissue reorganization in the nodule tissues of PI3k inhibitor-treated samples

**Figure 6:**
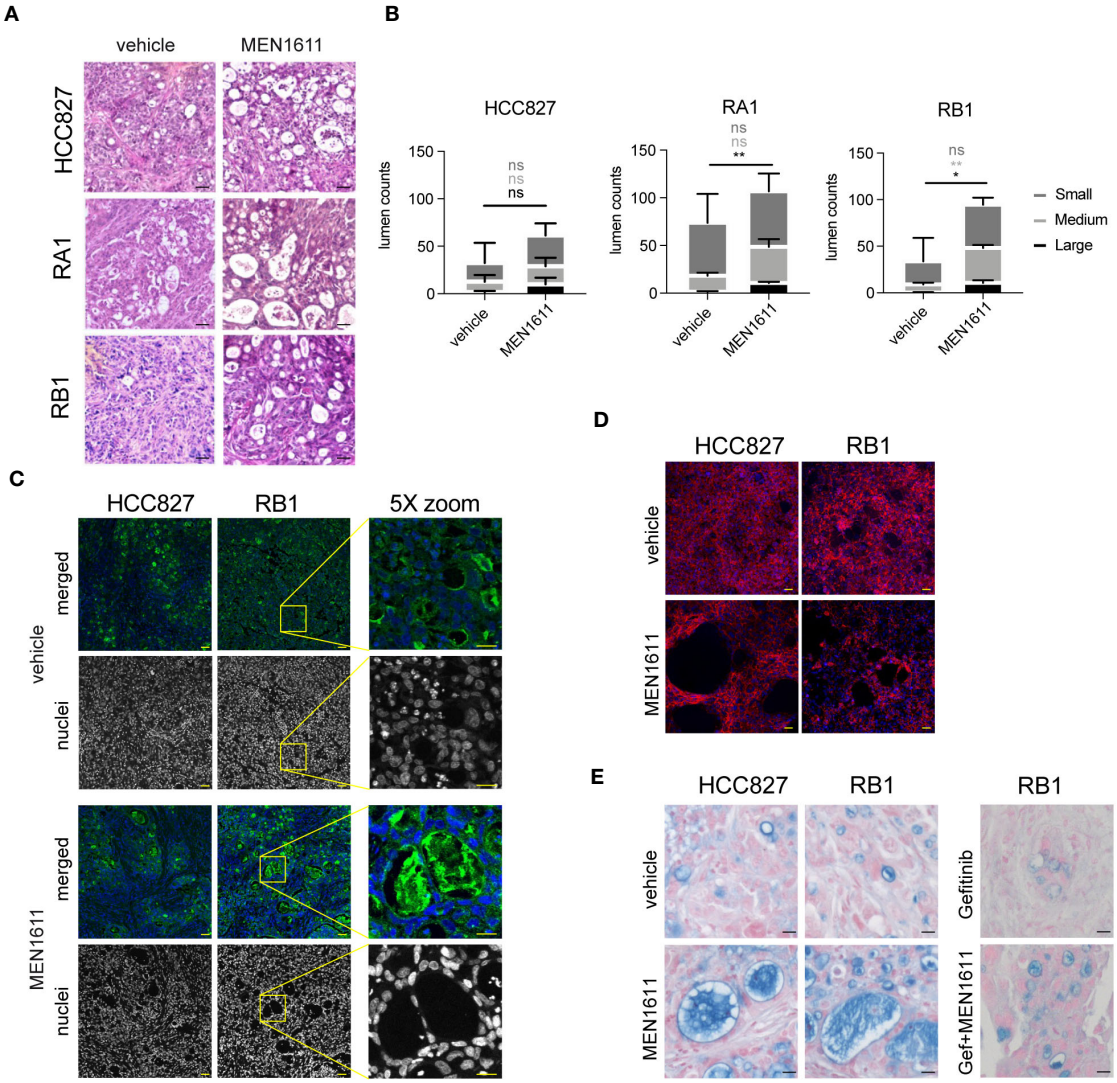
Tissue characterization of xenograft tumors after *in vivo* treatment. **(A)** Hematoxylin and eosin staining of paraffin sections from nodules of HCC827, RA1, RB1, and RA2 xenografts from mice treated with vehicle or MEN1611. Scale bar, 20 µm. **(B)** Lumen quantification was performed in 8–12 images captured by three distinct nodules per condition as described in Materials and Methods. For each lumen size (small ≤ 300 μm^2^; 300 μm^2^ < medium < 900 μm^2^; large ≥ 900 μm^2^), MEN1611 treatment was compared to the vehicle by unpaired t-test. Ns, not significant p-value, *p-value < 0.05, **p-value < 0.01. **(C)** Lectin-FITC and Hoechst 33342 staining (in merged pictures) or Hoechst 33342 staining alone (in grayscale picture) of HCC827 and RB1 xenograft nodule paraffin sections (×20 objective; confocal) from mice treated with vehicle and MEN1611. Scale bar, 50 µm. Depicted by the yellow box, a ×5 optical zoom of RB1 xenograft sample stained with lectin-FITC and Hoechst 33342 or with Hoechst33342 alone is shown. Scale bar, 25 µm. **(D)** E-Cadherin-647 staining of HCC827 and RB1 xenograft nodule paraffin sections (×20 objective; confocal) from mice treated with vehicle and MEN1611. Scale bar, 50 µm. **(E)** Alcian Blue Fast Red staining of HCC827 and RB1 xenograft nodule paraffin sections from mice treated with vehicle and MEN1611 and in RB1 samples, also with gefitinib and with the combination of MEN1611 and gefitinib. Scale bar, 20 µm.

Peculiar lumens in the nodule tissues were observed in H&E-stained samples of both sensitive and resistant cancer cell line xenografts from MEN1611-treated mice (**Figure 6A**). Remarkably, the number and area mean of the medium and large lumens were increased in all MEN1611-treated sample nodules and reached significant values in the RB1 and large lumen counts in the RA1 nodules (**Figure 6B**). Lumens are also often occupied by lectin positive materials staining glycoproteins, and sometimes, they contain murine nuclei (**Figure 6C**); moreover, cells surrounding the lumens are also positive for the epithelial marker E-Cadherin (**Figure 6D**). Alcian Blue/Fast Red staining revealed mucin presence in the lumens of both vehicle- and MEN1611-treated nodule samples (**Figure 6E**).

Intriguingly, lumens were also observed in monolayers of 2D cell cultures associated with cytoskeleton reorganization. HCC827 and RB1 cells, 48 h post-treatment with MEN1611, showed lumens and the presence of cortical actin and pseudopodia protrusions (**Figure S5**). The lumens are also present in other NSCLC cell lines treated with MEN1611, but not in the A549 and in the A431 vulvar cancer cell line (**Figures S5A, B**). Interestingly, gefitinib treatment was not associated with lumen formation in NSCLC. Overall, the morphological study in epithelial cancer cells indicates that MEN1611 increases lumen formation in cancer cell lines of different epithelial origins. Finally, spheroid 3D cultures were established to mimic the *in vivo* 3D growth of NSCLC cells in xenografts. Staining with phalloidin-TRITC and confocal microscopy analysis confirmed the formation of lumens in this 3D model upon treatment with MEN1611 (**Figure S5C**).

## Discussion

There is an urgent need to explore new ways to tackle progression and resistance in patients with EGFR activating mutations. In NSCLC, the PI3K/Akt/mTOR pathway has been implicated in both tumorigenesis and the progression of disease. Targeted therapies against this pathway have yielded disappointing results; clinical trials, however, have been mostly performed on molecularly unselected populations (23). Large studies of NSCLC patients by next-generation sequencing (NGS) identified *PIK3CA* gene mutations in 3.7% of patients, with predominance for squamous cell carcinoma (8.9%) compared with adenocarcinoma (2.9%). Importantly, oncogenic mutations in *PIK3CA* gene may coexist with mutations in gene encoding for EGFR, BRAF, anaplastic lymphoma kinase (ALK), and KRAS. Similar findings were obtained by Yamamoto et al. by examining NSCLC cell lines and NSCLC tumors for *PIK3CA* mutations in exon 9 or 20 and *PIK3CA* gene amplification (24). Literature data overall underline that only a small number of NSCLC patients carry mutations in the *PIK3CA* gene and could be considered suitable for a treatment—also in combination with other drugs—with a PI3KCA selective inhibitor. Here, we aim to investigate the effects of MEN1611 in models of NSCLC devoid of *PIK3CA* gene mutations and with EGFR activating mutations due to Exon19 deletion (Δ E746-A750) that are sensitive (HCC827) and resistant (RA1, RA2, RB1, RB1.1, and RB2) to EGFR-TKIs (erlotinib and gefitinib). The NSCLC cell model system is characterized by constitutive activation of targets of the PI3K signaling pathway (AKT and mTOR). Here, cellular and biochemical studies demonstrated that both sensitive HCC827 and HCC827-derived EGFR-TKI-resistant NSCLC cell lines show sensitivity to MEN1611 in the low micromolar range concentration. The inhibitor also suppresses the phosphorylation of selected targets of the PI3K/AKT/mTOR signaling pathways in all NSCLC cells. Moreover, we show that the PIK3CA mutational status did not influence the response to the PI3K inhibitor MEN1611 in NSCLC, suggesting the possibility of exploring combination therapy in patients with an EGFR mutation and constitutive activation of the PI3K/AKT/mTOR pathway. Interestingly, the HCC827 cell line showed a decrease in pAKT, p4EBP1, and pS6 upon treatment *in vitro* with either erlotinib or MEN1611. In contrast, in HCC827-derived EGFR-TKI-resistant cell lines, erlotinib does not suppress or only poorly suppresses the phosphorylation of the same PI3K/AKT/mTOR targets [ (5); this paper]. Overall, these data indicate that MEN1611 could be a candidate for the treatment of NSCLC with an erlotinib/gefitinib-resistant phenotype and constitutive activation of the PI3K/AKT/mTOR pathways, a phenotype mimicked by our model system. Our data indicate that selection on a functional basis in addition to a mutational gene profile may be beneficial for a drug treatment design. We also demonstrate that MEN1611 has a cytostatic effect *in vivo* in xenograft NSCLC tumors in immunodeficient mice and impairs cancer cell growth through inhibition of constitutively activated AKT and mTOR pathways. The *in vitro* and *in vivo* studies also showed a synergistic effect of erlotinib/gefitinib and MEN1611 in EGFR-TKI-resistant cells.

The contribution of the peculiar tissue architecture observed *in vitro* and *in vivo* to the anti-tumoral activity of the inhibitor should be further investigated as well as the signaling pathways involved. Interestingly, elegant studies by imaging single-cell ERK activity dynamics in mammalian MCF10A breast acini model systems demonstrated that cell fates during morphogenesis depend on spatio-temporal modalities of ERK pulse frequency (25). The study also reported that acini harboring oncogenic PIK3CA H1047R mutation, frequently mutated in breast cancer and leading to constitutive activation of the PI3K pathway, display increased ERK frequency with inner cell survival and absence of lumen formation. EGFR inhibition by gefitinib abolished ERK pulses in PIK3CA H1047R cells and the Pictilisib-mediated PI3K inhibition decreased ERK frequency in both wild-type and mutated cells (25). It is tempting to speculate that the PI3K/ERK crosstalk and the spatial regulation of ERK frequency might also play a role in the tissue architecture observed in the xenograft nodes of MEN1611-treated and MEN1611+gefitinib-treated mice, but this remains to be investigated.

Combination therapy is today a common strategy for the treatment of tumors to increase the success rate, improve the outcome and survival of patients, and avoid the selection of resistant cancer cells through the activation of compensatory pathways. It is interesting that the NSCLC cell lines resistant to EGFR-TKI respond to the PI3K inhibitor and that the increase of pERK1/2, likely induced by the activation of feedback loops, is controlled by gefitinib in the combination treatment.

## Conclusion

Overall, this preclinical study indicates that the PI3K δ-sparing inhibitor, MEN1611, could be a candidate for the treatment of NSCLC with an erlotinib/gefitinib-resistant phenotype and constitutive activation of the PI3K/AKT pathway.

## Data availability statement

The original contributions presented in the study are included in the article/**Supplementary Material**. Further inquiries can be directed to the corresponding authors.

## Ethics statement

Ethical approval was not required for the studies on humans in accordance with the local legislation and institutional requirements because only commercially available established cell lines were used. All animal procedures were performed in accordance with current guidelines of the European Ethical Committee (directive 2010/63/EU) and were in strict compliance with Italian Ministry of Health approved protocols (Authorizations N. 872/2015-PR, 898/2018-PR). The study was conducted in accordance with the local legislation and institutional requirements.

## Author contributions

GP: Conceptualization, Data curation, Formal Analysis, Investigation, Visualization, Writing – original draft, Writing – review & editing, Methodology. DP: Conceptualization, Data curation, Formal Analysis, Investigation, Methodology, Visualization, Writing – review & editing. VF: Data curation, Formal analysis, Investigation, Methodology, Visualization, Writing - review & editing. AP: Data curation, Formal Analysis, Investigation, Writing – review & editing, Visualization. CC: Formal Analysis, Investigation, Writing – review & editing. SA: Formal Analysis, Writing – review & editing, Methodology. FS: Investigation, Writing – review & editing. MR: Investigation, Writing – review & editing. GM: Investigation, Writing – review & editing, Methodology. MS: Investigation, Methodology, Writing – review & editing. MaB: Investigation, Methodology, Writing – review & editing. MoB: Funding acquisition, Investigation, Methodology, Writing – review & editing. GR: Conceptualization, Funding acquisition, Supervision, Data curation, Investigation, Formal analysis, Visualization, Writing – original draft, Writing – review & editing, Methodology. 
