# Supplementary figures and images for: The activity of a PI3K δ-sparing inhibitor, MEN1611, in non-small cell lung cancer cells with constitutive activation of the PI3K/AKT/mTOR pathway
Source: Front Oncol. 2023 Nov 14;13:1283951. doi: 10.3389/fonc.2023.1283951 (PMC10682785; doi:10.3389/fonc.2023.1283951)

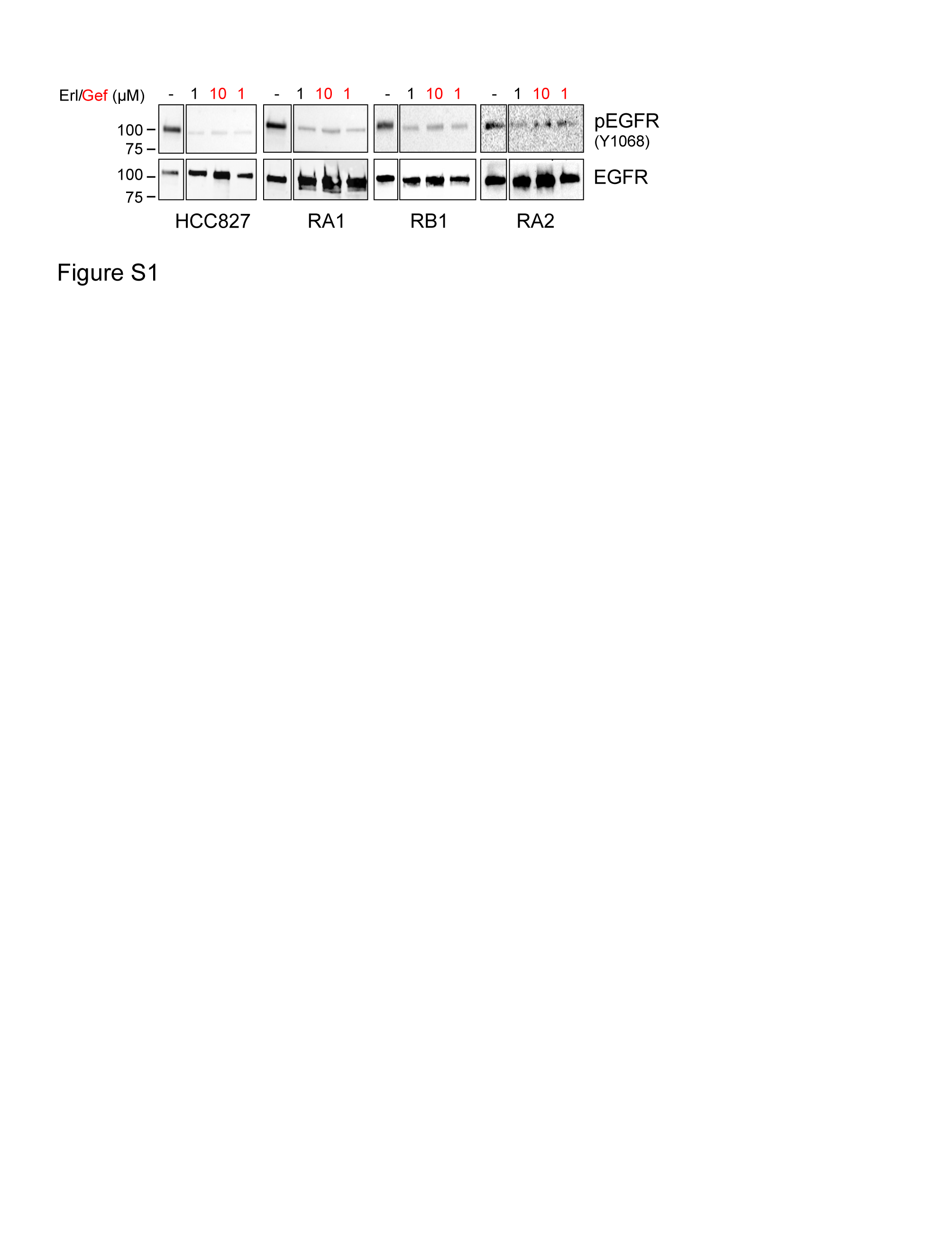

Supplement: Supplementary file 1 [file Image_1.tif]

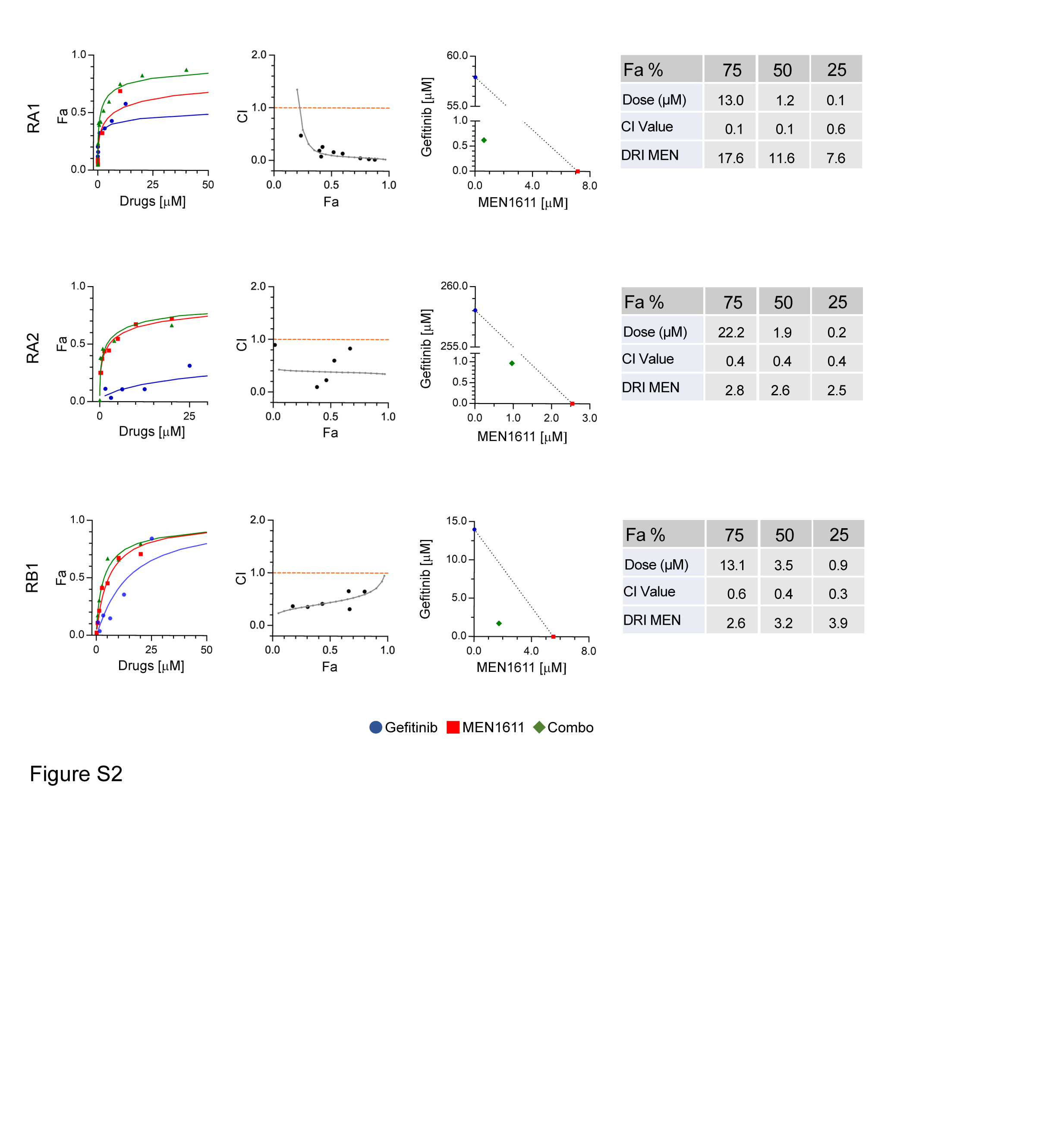

Supplement: Supplementary file 2 [file Image_2.tif]

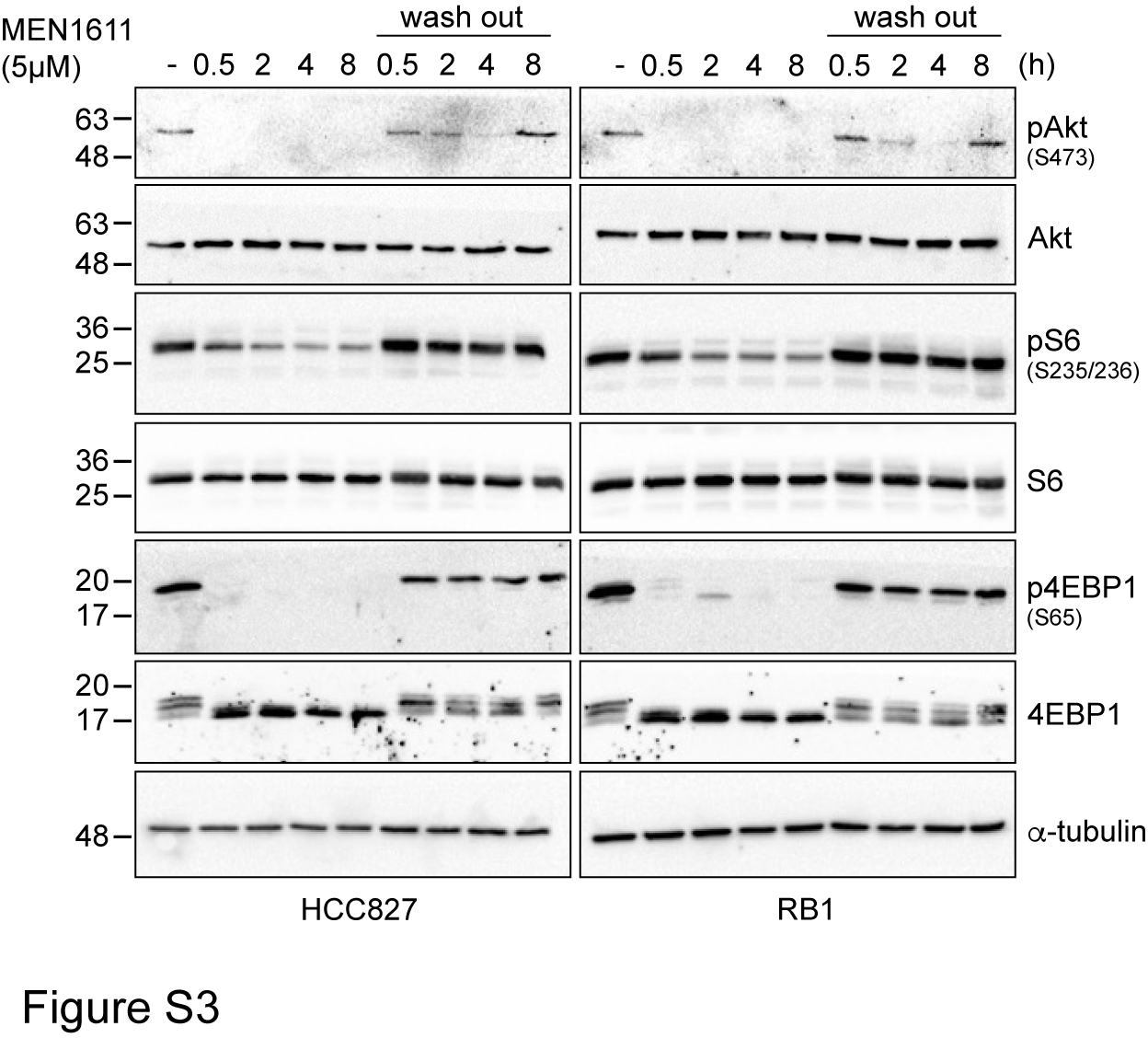

Supplement: Supplementary file 3 [file Image_3.tif]

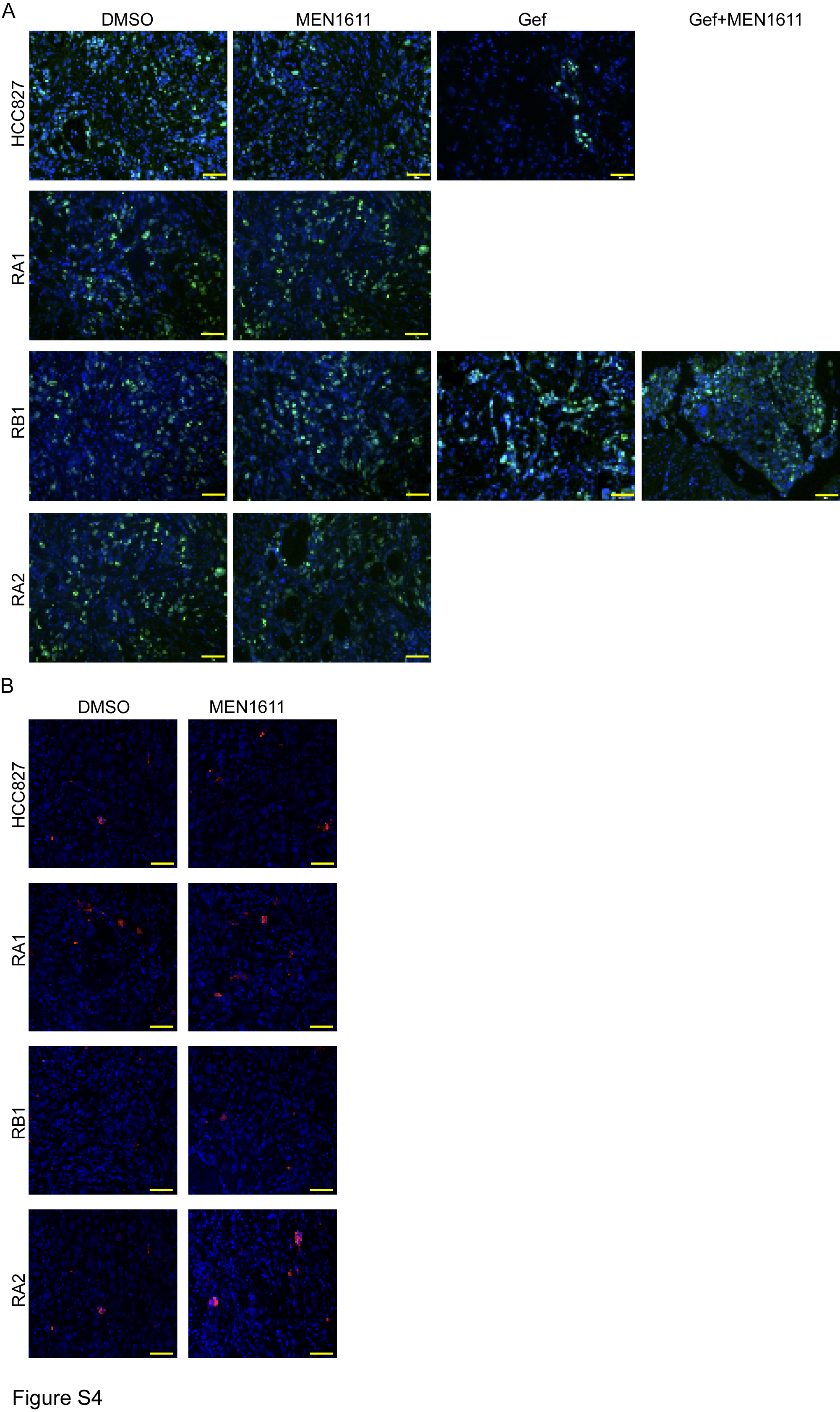

Supplement: Supplementary file 4 [file Image_4.tif]

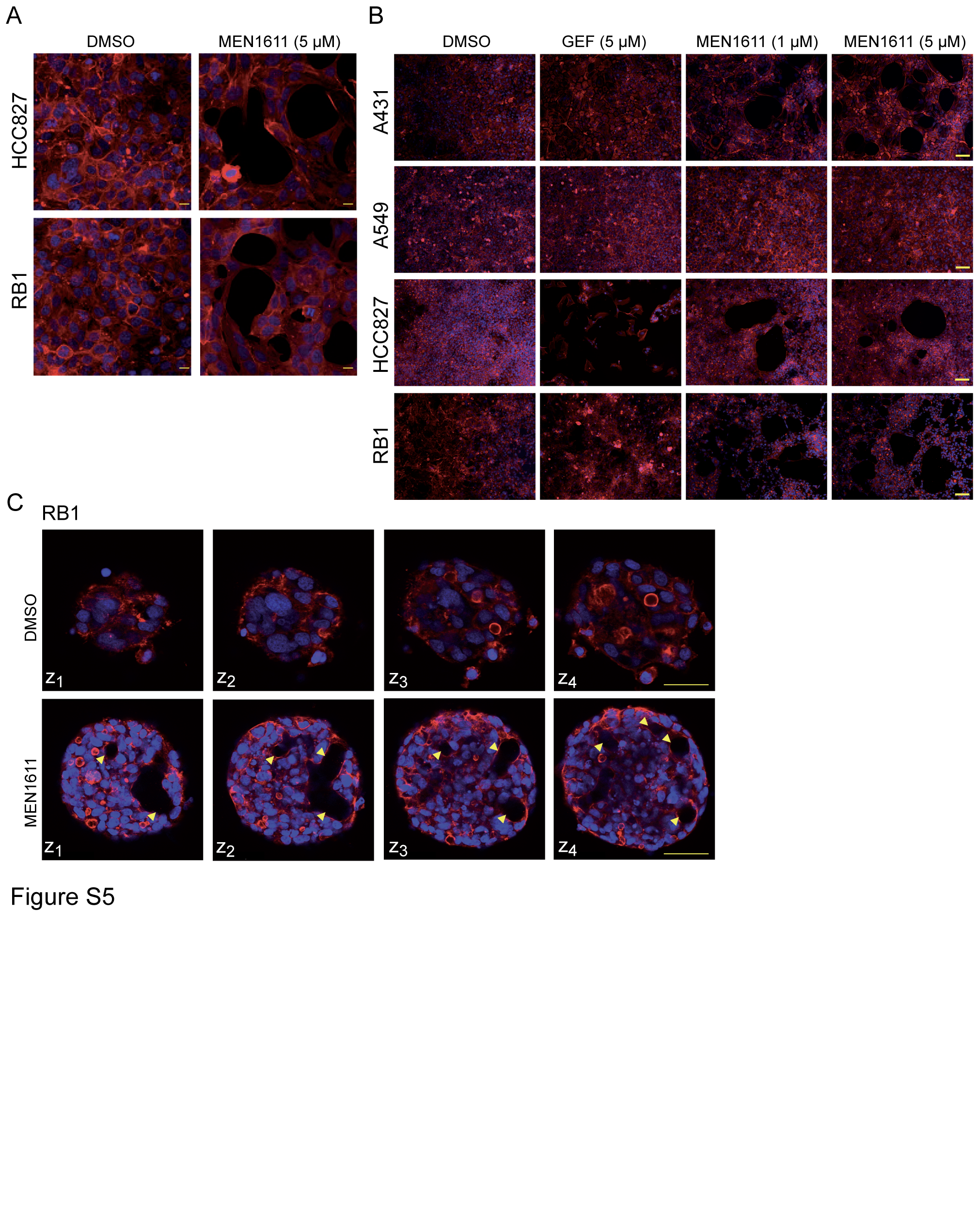

Supplement: Supplementary file 5 [file Image_5.tif]
