# Supplementary material for: The activity of a PI3K δ-sparing inhibitor, MEN1611, in non-small cell lung cancer cells with constitutive activation of the PI3K/AKT/mTOR pathway
Source: Front Oncol. 2023 Nov 14;13:1283951. doi: 10.3389/fonc.2023.1283951 (PMC10682785; doi:10.3389/fonc.2023.1283951)
Supplement: Supplementary file 6 [file Table_1.pdf]

Supplementary Material

The activity of a PI3K δ-sparing inhibitor, MEN1611, in Non-Small Cell Lung Cancer cells with a constitutive activation of the PI3K/AKT/mTOR pathway

Giuliana Papoff[1†]*, Dario Presutti[1†], Valentina Fustaino[1], Andrea Parente[1], Clelia Calandriello[1], Stefano Alemà[1], Ferdinando Scavizzi[1,2], Marcello Raspa[1,2], Giuseppe Merlino[2], Massimiliano Salerno[2], Mario Bigioni[2], Monica Binaschi[2] and Giovina Ruberti[1]*

* Corresponding authors:

Giuliana Papoff email: giuliana.papoff@cnr.it

Giovina Ruberti email: giovina.ruberti@cnr.it

Supplementary Figures and Tables

Table S1. Quantification of the percentage of events in sub-G1 (apoptotic), G0/G1, S, G2M cell cycle phases in cell lines treated with MEN1611 (5 µM) for 48 hours.

Cell line	Sub-G1(%)		G0/G1 (%)		S (%)		G2M (%)	
	DMSO	MEN1611	DMSO	MEN1611	DMSO	MEN1611	DMSO	MEN1611
**HCC827**	4 ± 2	15 ± 4*	40 ± 8	65 ± 3*	22 ± 7	18 ± 7	27 ± 14	14 ± 5
**RA1**	6 ± 1	14 ± 10	38 ± 4	67 ± 11*	21 ± 8	12 ± 2	26 ± 16	13 ± 6
**RB1**	5 ± 1	8 ± 3	49 ± 8	66 ± 3	22 ± 7	19 ± 8	24 ± 8	16 ± 7
**RA2**	7 ± 1	8 ± 4	44 ± 4	62 ± 5**	15 ± 4	14 ± 0	35 ± 8	22 ± 3

Table S2 . Exact p-values from statistical analysis of tumor nodules volumes

Table S3 . NSG mice weight and mortality of HCC827, RA1, RB1, RA2 xenograft murine models

Figure S1. Western blot analysis of pEGFR (Y1068)/EGFR in HCC827, RA1, RB1 and RA2 lysates from cells treated with Erlotinib (1 μM) (black) or Gefitinib (1,10 μM) (red). Basal phosphorylation levels correspond to lysates from cells treated with DMSO (-).

Figure S2. Dose-response analysis of the combination treatment with Gefitinib and MEN1611. Dose effect curves, Combination Index (CI) plots and isobologram plots of RA1, RA2 and RB1 NSCLC cell lines treated with Gefitinib (blue), MEN1611 (red) or Combo (Gefitinib/MEN1611) (green) at 1:1 ratio are shown.

Figure S3. Western blot analysis of cell protein lysates. Cells treated with MEN1611 at 5 μM for 2 h were collected at 30', 2h, 4h and 8h or washed and then collected at the same time points. PI3K/AKT pathway was analyzed by using the indicated antibodies. Tubulin was used as a normalizer.

Figure S4. Ki67 and cleaved Caspase-3 immunostaining. Immunostaining of xenograft nodule sections of the murine models indicated with: A) Ki67 antibody/anti-rabbit Alexa 488; B) Cleaved Caspase 3 antibody/anti-rabbit Alexa 546 (20X objective). Scale bar: 50 μm.

Figure S5. The cytoskeleton organization in MEN1611-treated 2D and 3D cultures. Phalloidin-TRITC staining of A) HCC827 and RB1 cell lines treated with DMSO and MEN1611 at 5 μM for 48 h (20X objective, zoom 3X). Scale bar: 15 μm; and of B) A431, A549, HCC827 and RB1 cells treated with DMSO, Gefitinib (5 μM) or MEN1611 (1-5 μM) for 48 h. Nuclei are stained with Hoechst 33342; C) Confocal Z stacks (5 μM depth) images of RB1-spheroids treated with DMSO or MEN1611 (10 μM) for 48 h. Yellow arrows indicate lumens path in the spheroid depth. Scale bars in b and c: 50 μm.
